# Unveiling the Uncommon: A Case Series on Cushing’s Disease During Pregnancy

**DOI:** 10.7759/cureus.66045

**Published:** 2024-08-02

**Authors:** Ravi Shah, Subin S, Vanita Suri, Sanjay K Bhadada, Rama Walia

**Affiliations:** 1 Endocrinology, Postgraduate Institute of Medical Education and Research, Chandigarh, IND; 2 Obstetrics and Gynecology, Postgraduate Institute of Medical Education and Research, Chandigarh, IND

**Keywords:** suppression test, striae, cushing disease, pregnancy, cushing syndrome

## Abstract

Cushing's disease (CD) is characterized by a high rate of hypogonadism and infertility. It is difficult to detect CD in pregnant women who present with symptoms such as weight gain, striae, headache, backache, and pedal edema that overlap with the physiological changes of pregnancy. In this paper, we present three cases of CD likely missed during pregnancy and later also discuss the approach to diagnosis and management of CD in pregnancy.

## Introduction

In females, cortisol exerts its effects at multiple levels within the hypothalamic-pituitary-gonadal axis. At the hypothalamic level, cortisol inhibits the secretion of gonadotropin-releasing hormone. At the pituitary level, it suppresses the release of luteinizing hormone and follicle-stimulating hormone. At the gonadal level, cortisol impairs steroidogenesis and gametogenesis. These disruptions may contribute to conditions such as hypogonadism, polycystic ovary syndrome (PCOS), and infertility [1,2].

Diagnosing Cushing's disease (CD) in pregnant women can be difficult due to the overlap between its symptoms such as weight gain, striae, headache, backache, pedal edema, and the typical physiological changes of pregnancy. In this paper, we describe three cases of CD that were potentially missed during pregnancy and discuss strategies for diagnosing and managing CD in pregnant patients. Pregnancy-associated CD is defined as its onset during gestation or within 12 months of delivery or miscarriage [3]. The overlapping nature of CD symptoms with typical pregnancy may lead to missed diagnoses during this critical period [4].

Our case studies, presented at the Department of Endocrinology, Postgraduate Institute of Medical Education & Research (PGIMER), Chandigarh, between January 2020 and January 2023, highlight this issue and offer valuable insights into managing CD during pregnancy.

## Case presentation

Case 1

**Figure 1:**
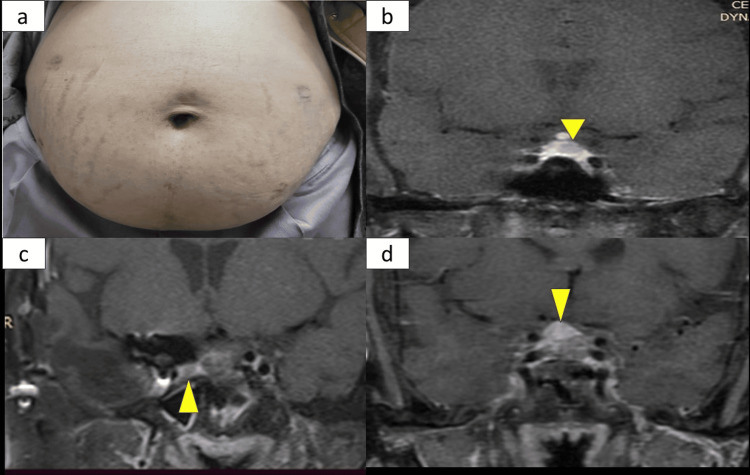
(a) Case 1: Thin purplish striae over the patient's abdomen. (b) Case 1: post-contrast T1 weighted MRI image suggestive of pituitary microadenoma (yellow arrow). (c) Case 2 post-contrast T1 weighted CEMR of pituitary and hypothalamic area suggestive of residual pituitary microadenoma (yellow arrow) with distorted sellar anatomy due to previous surgery. (d) Case 3: Post-contrast T1-weighted CEMR pituitary and hypothalamic area suggestive of pituitary macroadenoma (yellow arrow) CEMR: Contrast-enhanced magnetic resonance imaging

A 29-year-old female initially presented during pregnancy at 12 weeks of gestation (POG) and was diagnosed with hypertension (HTN). The patient had a normal oral glucose tolerance test (OGTT) in the first trimester. Still, it was found to be deranged at 28 weeks, indicating the development of gestational diabetes mellitus (GDM) for which the patient received insulin. At 32 weeks POG, the patient underwent a cesarean section due to non-progression of labor. Uncontrolled hyperglycemia was observed during delivery, and the patient received an insulin infusion. Two months post-delivery, the patient's relatives noticed bilateral lower limb weakness and psychiatric symptoms like anxiety and suicidal thoughts. These symptoms were associated with purple striae (Figure 1a), facial plethora, monomorphic acne, bilateral pedal edema, and easy fatigability.

Under the suspicion of CD, the patient was referred from gynecology to endocrinology for further evaluation. Baseline plasma adrenocorticotrophic hormone (ACTH) and cortisol levels were elevated, ruling out exogenous Cushing's syndrome (CS) (ACTH-144 pg/ml and 287 pg/ml, cortisol 1459 mmol/L). Elevated late-night plasma cortisol (11 p.m.) suggested a loss of diurnal variation (1655 nmol/L). Plasma dehydroepiandrosterone sulfate (DHEAS) was also elevated (456 mcg/dl). The overnight dexamethasone suppression test (ONDST) and low-dose dexamethasone suppression test (LDDST) were non-suppressible (1830 and 1760 nmol/L, respectively) (Table 1).

**Table 1:** Biochemical investigations on presentation ACTH: Adrenocorticotrophic hormone; ONDST: overnight dexamethasone suppression test; LDDST: low-dose dexamethasone suppression test; HDDST: high-dose dexamethasone suppression test

Test	Case 1	Case 2	Case 3	Reference range
8 AM Cortisol (nmol/L)	1459	457	972	171-536
8 AM ACTH (pg/ml)	144	63	45.9	7.2-63.3
11 PM Cortisol (nmol/L)	1655	313	970	<207
ONDST (nmol/L)	1830	313.5	878	<50
LDDST (nmol/L)	1760	80.1	454	<50
HDDST (% decline as compared to baseline 8 AM cortisol)	55.17%	90.3%	80.3%	>50%

The workup indicated endogenous CS, which was ACTH-dependent. Contrast-enhanced magnetic resonance imaging (CEMR) of the pituitary and hypothalamic area revealed a 4-5 mm pituitary microadenoma in the left half of the pituitary (Figure 1b). Contrast-enhanced computed tomography (CECT) chest/abdomen/pelvis and gallium DOTATATE PET scan of the whole body did not suggest any lesions or uptake elsewhere, making ectopic CS less likely. Additionally, the patient had uncontrolled hyperglycemia (HbA1c=8.2%), secondary hypothyroidism, and hypogonadism.

The patient developed a lower respiratory tract infection and delirium during admission, leading to an emergency laparoscopic total bilateral adrenalectomy. Subsequently, the patient was treated with antibiotics and discharged with a plan for definitive management of the pituitary adenoma in the follow-up.

Case 2

A 28-year-old female presented with severe headache, decreased vision, and ptosis one month after childbirth. Following this episode, she experienced lactational failure and developed secondary amenorrhea, which is persistent even today, 10 years post-delivery. Despite being lost to follow-up, her ptosis spontaneously improved, but her vision did not. After four years of delivery, she had a recurrence of ptosis, worsened vision, and intensified headache. Notably, she exhibited Cushingoid features in the form of broad purple striae (Figure 1a), facial plethora, and proximal myopathy, prompting evaluation by an endocrinologist.

Laboratory investigations revealed the following parameters: 11 pm cortisol - 313nmol/L, ONDST - 313.5nmol/L, LDDST - 80.1 nmol/L, high-dose dexamethasone suppression test (HDDST) - 43.9 nmol/L, ACTH - 63 pg/ml, DHEAS - 121.4 mcg/dl, prolactin - 3 ng/ml, TSH - 1.7 mcIu/ml, T3 - 1 ng/ml, and T4 - 4.6 mcg/dl (Table 1).

CEMR of the pituitary and hypothalamic area revealed a pituitary macroadenoma with apoplexy; hence, the patient underwent endoscopic transsphenoidal surgery. Histopathology confirmed the presence of a pituitary adenoma that was positive for ACTH on immunostaining. Moreover, the Kiel 67 (Ki67) index was greater than 20%. Post-operatively, on day 3, the patient had morning cortisol of 280 nmol/l, suggesting that the patient did not go into biochemical remission. Cortisol dynamics were reassessed six months later, and the patient was found to have persistent CD with imaging suggestive of residual macroadenoma. She underwent transfrontal surgery but did not achieve biochemical remission. She underwent 30 fractions of external beam radiotherapy (EBRT) (54 Gray) in 2020, but the disease persisted. Consequently, six years post-delivery, the patient was started on temozolomide and cabergoline. A combination of EBRT and temozolomide led to the normalization of her 24-hour urinary-free cortisol (UFC) eight years post-delivery, after which temozolomide was discontinued.

The patient remains eucortisolic, but the structural disease persists as a 9*3*3 mm residual in the right side of the Sella (Figure 1c). Till the last follow-up of the patient three months ago, there has been no interval growth observed six months after stopping temozolomide.

Case 3

A 35-year-old female presented with a history of weight gain, hair thinning, and hirsutism for the past 10 years. Initially, she was diagnosed with PCOS. However, over the next two years, she developed prominent features of CS, including proximal weakness, abdominal obesity, striae, and hyperpigmentation. Her biochemical investigations were as follows: 8 am cortisol = 972 nmol/L, ACTH = 45.9 pg/ml; 11 pm cortisol = 970 nmol/L, with non-suppressible ONDST = 878 nmol/L, and HDDST = 191 nmol/L (Table 1). A contrast-enhanced MRI of the pituitary and hypothalamic area revealed a 5*7 mm pituitary microadenoma in the left half of the pituitary gland. The patient underwent transsphenoidal surgery, and on post-operative day 3, serum cortisol was 482 nmol/L. The patient became pregnant one-month post-surgery and was lost to follow-up during pregnancy. She was documented to have gestational HTN and diabetes mellitus, complicating pregnancy. She delivered a preterm female child after a cesarean section. However, there was no history of lactational failure.

Three years after delivery, the patient experienced a recurrence of symptoms, including progressive weight gain and oligomenorrhea. She presented with 8 am cortisol = 827 nmol/L, ONDST = 800 nmol/L, LDDST = 413 nmol/L, and ACTH = 34.4 pg/ml. The repeat contrast-enhanced MRI of the pituitary and hypothalamic area suggested a 9*5*9mm pituitary microadenoma, indicating a possibility of persistent CD. Considering the recurrence of symptoms and the unwillingness of the patient to opt for surgery, the patient was started on ketoconazole initially at 400mg/day, which was up-titrated to 800mg/day based on 24-hour urinary-free cortisol levels.

In follow-up, the patient agreed to surgery, and her cortisol dynamics were reassessed. Following the cessation of ketoconazole, her cortisol dynamics were reassessed: 11 pm cortisol = 412 nmol/L, ONDST = 257 nmol/L, LDDST = 188 nmol/L, and ACTH = 24 pg/ml. Contrast-enhanced MRI of the pituitary and hypothalamic area revealed a pituitary macroadenoma of 11*9*6mm (Figure 1d), while Gallium DOTATE PET CT showed no peripheral lesion. The patient underwent endoscopic transsphenoidal surgery with near-total excision, and her post-operative course was uneventful. She experienced a weight loss of 2 kg, and her insulin requirement was reduced from a daily dose of 80 U to 20 U/day, allowing her to discontinue antihypertensive medications. On post-operative day 3, cortisol was 177 nmol/L, and the patient is currently in follow-up with plans for a repeat MRI after six months.

## Discussion

Regardless of its source, hypercortisolism during pregnancy poses significant risks to both the mother and the fetus. Pregnancy in the context of CD is exceedingly rare due to the potential impact of hypercortisolism on the hypothalamo-pituitary gonadal axis, leading to hypogonadism and infertility. Among pregnancy-related cases, approximately 60% arise from adrenal origin, which contrasts with non-pregnant women, where CD is implicated in about 70% of cases. This discrepancy in etiology patterns is likely due to the lower incidence of suppression of the hypothalamic-pituitary-adrenal (HPA) axis in adrenal CS [5].

Pregnancy triggers the maternal HPA axis activation, resulting in increased circulating ACTH, free and total cortisol, and 24-hour urinary free cortisol. Additionally, during pregnancy, the placenta produces significant amounts of CRH (corticotropin-releasing hormone) and related peptides, escalating exponentially in the third trimester. The placental enzyme, 11β-hydroxysteroid dehydrogenase type 2, also converts cortisol to cortisone. As a result, plasma cortisol concentration rises during pregnancy while maintaining its diurnal variation [4].

Symptoms such as weight gain, striae, headache, backache, and pedal edema are typical during pregnancy. However, specific features should raise suspicion for CS, which is not usual or physiological in pregnancy. These include broader and more purple striae, striae appearing at unusual sites, skin thinning, easy bruisability, a tendency for spontaneous fractures, and proximal myopathy [4].

Pregnancy in CS should be avoided as it is associated with the following complications (Table 2) [6,7].

**Table 2:** Maternal and fetal complications with Cushing’s syndrome in pregnancy

Maternal	Fetal
Pre-term deliveries	Deaths
Gestational diabetes mellitus	Pre-term births
Hypertension, pre-eclampsia	Neonatal infections
Higher rates of cesarean section	Hypoglycemia
	Respiratory distress

Cases 1 and 3 highlight the impact of hypercortisolism during pregnancy, leading to the development of GDM and HTN, ultimately requiring cesarian sections for delivery. Case 3 experienced an additional challenge with preterm birth.

During pregnancy, the 1 mg ONDST may yield false positive results, with about 60% of normal pregnant women unable to suppress cortisol levels below 50 nmol/L (1.8 μg/dL). This lack of suppression is due to the blunting effect of dexamethasone on cortisol levels during pregnancy. Another marker used to assess cortisol levels is the UFC excretion, which increases up to three-fold during pregnancy. UFC values higher than the pregnancy-specific threshold can aid in diagnosing CS. The circadian rhythm of cortisol is preserved during pregnancy, making late-night salivary cortisol or midnight serum cortisol levels useful for diagnostic testing. The ELISA-Cortisol EIA kit (salimetrics) suggests specific cut-offs for different trimesters to achieve diagnostic accuracy with good sensitivity (80%) and specificity (93%) [8].

Pituitary MRI without gadolinium is generally considered safe during pregnancy, but abdominal MRI is preferred to characterize the mass better following the initial screening with ultrasound imaging for adrenal CS.

Literature on the treatment of endogenous CS during pregnancy, regardless of the source, is limited, with fewer than 100 case reports available. Treatment approaches include surgery (24%), medical treatment, which includes metyrapone (11%), or a combination of both (4.7%) [9,10]. For mild cases or those diagnosed later in pregnancy, conservative management may involve addressing comorbidities.

It may take one month or more to restore normal dexamethasone suppressibility of cortisol post-delivery, and CBG may remain elevated for three months postpartum. Hence, disease reassessment should be deferred to 2-3 months post-partum. This is also illustrated in Case 1, where ONDST and cortisol dynamics as early as three months postpartum clinched the diagnosis of CD [7].

Breastfeeding is not contraindicated; however, it may not be possible due to lactational failure secondary to CS or the effect of medical treatment like cabergoline [7].

Hence, a summary of the approach to CD in pregnancy is given in Table 3.

**Table 3:** Summary of approach to Cushing's syndrome in pregnancy

Parameter	Important points to consider
Clinical suspicion	Development of striae at sites other than the abdomen
	Proximal myopathy
	Fragility fractures
	Easy bruising
	Skin thinning
	Hypertension
	Hyperglycemia
Diagnosis	24 hour UFC > 3* ULN
	Late-night salivary cortisol: > 0.255 μg/dL (7.0 nmol/L) for the first trimester 0.260 μg/dL (7.2 nmol/L) for the second trimester 0.285 μg /dL (7.9 nmol/L) for the third trimester
Source localization	ACTH dependent: Plain MR pituitary and hypothalamic area
	ACTH independent: Plain MRI of the abdomen
Treatment	Mild, no comorbidities: Observe
	Moderate to severe, with comorbidities: surgery- in the second-trimester medical management- metyrapone, ketoconazole

## Conclusions

CS during pregnancy is frequently overlooked despite its potential to cause severe complications for both the mother and the fetus. Therefore, it is crucial to maintain a high level of suspicion for CS in pregnant individuals, particularly if they exhibit striae at sites other than the abdomen, proximal myopathy, fragility fractures, easy bruising, skin thinning, hypertension, and hyperglycemia. Prompt recognition and diagnosis of the condition are essential to initiate appropriate management.

However, diagnosing and managing CS in pregnancy is challenging, even after a high index of clinical suspicion. Therefore, as discussed earlier, a comprehensive and well-informed approach to diagnosis, evaluation, and treatment should be adopted. Early detection and appropriate management are paramount in ensuring the well-being of both the mother and the developing fetus. Timely intervention and multidisciplinary care are vital to achieve the best possible results for these complex cases.
